# A multi-state evaluation of extreme risk protection orders: a research protocol

**DOI:** 10.1186/s40621-024-00535-z

**Published:** 2024-09-09

**Authors:** April M. Zeoli, Amy Molocznik, Jennifer Paruk, Elise Omaki, Shannon Frattaroli, Marian E. Betz, Annette Christy, Reena Kapoor, Christopher Knoepke, Wenjuan Ma, Michael A. Norko, Veronica A. Pear, Ali Rowhani-Rahbar, Julia P. Schleimer, Jeffrey W. Swanson, Garen J. Wintemute

**Affiliations:** 1 Institute for Firearm Injury Prevention, School of Public Health, University of Michigan, 1415 Washington Heights, Ann Arbor, MI 48109 USA; 2 The Johns Hopkins Bloomberg School of Public Health, 415 N. Washington, Baltimore, MD 21205 USA; 3 New Jersey Gun Violence Research Center, School of Public Health, Rutgers University, 683 Hoes Lane, Piscataway, NJ 08854 USA; 4 Department of Emergency Medicine, University of Colorado, 12505 E. 16th Ave, Aurora, CO 80045 USA; 5 College of Behavioral and Community Sciences, University of South Florida, 4202 E. Fowler Avenue, Tampa, FL 33612 USA; 6 Department of Mental Health and Addiction Services, School of Medicine, Yale University, 333 Cedar Street, New Haven, CT 06510 USA; 7 University of Colorado Anschutz Medical Campus, 13001 East 17th Place, Aurora, CO 80045 USA; 8 College of Social Science, Michigan State University, 509 East Circle Drive, East Lansing, MI 48824 USA; 9 Davis School of Medicine, University of California, 2315 Stockton Blvd., Sacramento, CA 95817 USA; 10 School of Public Health, University of Washington, 3980 15th Avenue NE, Box 351616, Seattle, WA 98195 USA; 11 Duke University School of Medicine, 2400 Pratt Street, Box 102505, Durham, NC 27705 USA

**Keywords:** Extreme risk protection order, Data management, Secondary trauma, Data abstraction

## Abstract

**Background:**

Extreme Risk Protection Orders (ERPOs) are civil court orders that prohibit firearm purchase and possession when someone is behaving dangerously and is at risk of harming themselves and/or others. As of June 2024, ERPOs are available in 21 states and the District of Columbia to prevent firearm violence. This paper describes the design and protocol of a six-state study of ERPO use.

**Methods:**

The six states included are California, Colorado, Connecticut, Florida, Maryland, and Washington. During the 3-year project period (2020–2023), ERPO case files were obtained through public records requests or through agreements with agencies with access to these data in each state. A team of over four dozen research assistants from seven institutions coded 6628 ERPO cases, abstracting 80 variables per case under domains related to respondent characteristics, events and behaviors leading to ERPO petitions, petitioner types, and court outcomes. Research assistants received didactic training through an online learning management system that included virtual training modules, quizzes, practice coding exercises, and two virtual synchronous sessions. A protocol for gaining strong interrater reliability was used. Research assistants also learned strategies for reducing the risk of experiencing secondary trauma through the coding process, identifying its occurrence, and obtaining help.

**Discussion:**

Addressing firearm violence in the U.S. is a priority. Understanding ERPO use in these six states can inform implementation planning and ERPO uptake, including promising opportunities to enhance safety and prevent firearm-related injuries and deaths. By publishing this protocol, we offer detailed insight into the methods underlying the papers published from these data, and the process of managing data abstraction from ERPO case files across the multi-state and multi-institution teams involved. Such information may also inform future analyses of this data, and future replication efforts.

**Registration:**

This protocol is registered on Open Science Framework (https://osf.io/kv4fc/).

**Supplementary Information:**

The online version contains supplementary material available at 10.1186/s40621-024-00535-z.

## Background

In 2022 in the United States, over 27,000 people died by firearm suicide and more than 20,000 people were killed as a result of interpersonal firearm violence resulting in 14.2/100,000 people dying from intentional firearm injuries that year (Centers for Disease Control and Prevention, National Center for Injury Prevention and Control 2023). Preventing firearm access by those identified to be at risk of harming themselves and/or others is a logical strategy to reduce firearm homicide, firearm suicide, and nonfatal firearm violence. One promising and innovative opportunity to address firearm violence, therefore, is with extreme risk protection order (ERPO) laws. ERPO laws, or “red flag laws” as they are often called in popular discourse, provide a civil court process to temporarily prohibit firearm purchase and possession by individuals who are behaving dangerously and are at risk of harming themselves or others. As of June 2024, 21 states and the District of Columbia have passed ERPO-style bills into law (Johns Hopkins Bloomberg School of Public Health 2024). ERPOs fill an important policy gap because some individuals at risk of harming themselves and/or others, are legally able to purchase and possess firearms and cannot otherwise be disarmed. Therefore, ERPOs provide a mechanism for preventing firearm access (and potentially firearm violence) when an individual who represents a credible threat of violence is known but is not prohibited from accessing firearms by other legal mechanisms.

Research on ERPOs and their use and outcomes is in its infancy. Multiple studies have described characteristics of ERPO respondents and risk behaviors detailed in the applications in a single state or county ERPO (Barnard et al. 2021; Frattaroli et al. 2020; Pear et al. 2022; Rowhani-Rahbar et al. 2020; Swanson et al. 2019, 2017; Zeoli et al. 2021). Few studies have examined outcomes, and those that have generally focus on suicide outcomes, with findings suggestive of a reduction in suicide risk when ERPOs are used (Swanson et al. 2019, 2017; Miller et al. 2024) and an association at the state-level between ERPO law enactment and a reduction in firearm suicides (Kivisto and Phalen 2018). To our knowledge, this is the first multi-state ERPO study.

Here we describe the protocol we used to conduct a six-state study of ERPO case files designed to characterize ERPO petitions, petitioners and respondents (individual parties in the ERPO petition), court outcomes, and identify whether ERPOs are associated with reductions in suicide across geographically, demographically, and politically diverse states. The protocol described in this manuscript details (1) how we accessed ERPO case files in six states; (2) an explanation of the process of standardizing data from official records across the six states; (3) guidance for training research assistants (RAs) and maintaining consistent data abstraction practices across a multi-state, multi-institution RA team; and (4) strategies for reducing and responding to secondary trauma risk experienced by RAs as a result of reading ERPO narratives, which can include graphic descriptions of violence and crises.

## Methods

### Sample

During the 3-year project period (2020–2023), we conducted a multi-state study (Zeoli et al. 2022) of ERPO use with data from six states (California, Colorado, Connecticut, Florida, Maryland, and Washington). We selected these states for three reasons. First, all are engaged in efforts to implement ERPOs, and those implementation efforts are either yielding a critical mass of ERPO petitions filed or an informative implementation context. Second, these states are geographically and politically diverse, which may impact implementation and use. Third, we were able to access ERPO case files in the selected states. While ERPO statutes differ in some ways across the six states (Smart et al. 2020), all share a general process that involves a petition, court hearing, and court decision about whether to temporarily prohibit the individual named in the order from purchasing and possessing firearms.

ERPO court records are publicly available for all study states except Maryland. In the five states where ERPO data are public, we requested ERPO court records through public records searches or through agencies with access to these data. For California and Washington, ERPO case numbers and non-public identifying information such as respondent name, county, and ERPO date and type were first obtained from the Department of Justice (DOJ) (for California) and the Administrative Office of the Courts (for Washington) through a special request; this information was then used to request the publicly available court records from individual local and county courts throughout the two states.

In Colorado, a local team member contacted each county court to request ERPO records. In Connecticut, the ERPO statute (Connecticut General Assembly 2023) specifically requires the court to give notice of the court order to the Department of Mental Health and Addiction Services, and it is through these court notices (that have been maintained since 2013) that the study team accessed the public records. In Florida, we obtained most of the case files through Florida’s secure Comprehensive Case Information System (CCIS), a centralized database of court case information, which streamlined the process of accessing these publicly available records. For a few counties, we obtained the publicly available case files directly from the County Clerks of Court.

In Maryland, at the time of the study, ERPO records were restricted to select entities named in the statute (Brown 2022). Working with the Maryland Attorney General’s Office, we requested and obtained ERPO case files from District Courts throughout the State.

It should be noted that ERPO court records are often paper documents and may not be digitally accessible. This is true for California, Maryland, and Washington. Accessing paper copies of ERPO case files in these three states required a significant amount of time and coordination to collect the documents and scan and upload them to secure, password protected file storage systems housed at the collaborating universities in each state. The study teams in Colorado, Connecticut, and Florida gained access to digital copies of case files.

We requested ERPO case files for the time period beginning at ERPO enactment in each state through June 30, 2020 (see Table 1) with the exception of Connecticut, where the law took effect in 1999 but full ERPO case reports were only available beginning January 1, 2013. For California, the request process differed slightly. We first obtained identifying information on ERPO respondents through California DOJ and used that information to request the publicly available case files. However, due to California DOJ’s process of overwriting respondents’ older orders with newer orders in the primary file every 3 weeks, it is possible that, in the early days of collecting California’s ERPO case files, we missed cases when an individual was a respondent to more than one ERPO action. Once the California team learned of the California DOJ process in mid-2019, we started requesting ERPO case numbers and respondent identifying information from the California DOJ every 21 days so that we would not miss any order data due to the data overwriting process.

**Table 1:** States included in the study, and ERPO files coded, record obtainment, and data quality and fidelity

State	Inclusion start date of cases in study	Number of months in the study	Number of cases filed	Number of cases coded	Date of ERPO enactment	ERPO court record access	Method of obtaining records	Record copy	RA training
California	1/1/2016	54	1483*	961*	1/1/2016	Public	ERPO case numbers and non-public identifying information obtained from Department of Justice through a special request; this information was then used to request publicly availablethe court records from individual local and county courts	Paper and digital	Didactic training Practice coding Synchronous sessions
Colorado	1/1/2020	6	52	52	1/1/2020	Public	Local team member contacted each county court to request ERPO records	Digital	Didactic training Practice coding Synchronous sessions
Connecticut	1/1/2013	90	1407	1407	6/29/1999	Public	ERPO statute requires the court to give notice of court order to the Department of Mental Health and Addiction Services. The study team accessed the records through these court notices	Digital	Didactic training Practice coding State-specific coding manual instructing RAs where to find data elements in case files
Florida	3/18/2018	26	4695	2406	3/9/2018	Public	Most ERPO case files were accessed through Florida’s secure Comprehensive Case Information System (CCIS). A minority of Obtained a few case files were obtained directly from County Clerks of Court where CCIS access was restricted =	Paper and dDigital	Didactic training Practice coding One synchronous sessions
Maryland	10/1/2018	21	1347	1347	10/1/2018	Restricted	Working with the Maryland Attorney General’s Office, the study team requested and obtained ERPO case files from District Courts throughout the State	Paper	Didactic training Practice coding One synchronous session
Washington State	12/8/2016	43	455	455	12/08/2016	Public	ERPO case numbers and non-public identifying information obtained from the Administrative Office of the Courts through a special request; this information was then used to request the court records from individual local and county courts	Paper and digital	Synchronous training sessions Standing weekly meetings
Total		240		6628					

*In California, these numbers reflect the number of respondents, not cases

This effectively means we were unable to get case-level data for California prior to mid-2019 and therefore cannot distinguish the number of cases filed. Instead, the California data reflects the number of respondents from ERPO enactment through June 30, 2020, and the number of respondents for whom we coded cases for that timeframe. Additionally, in California, we received few requested case files from the court for cases involving only emergency ERPOs (i.e., those not followed by a temporary or final order) because these orders are granted remotely while the petitioning officer is in the field. As a result, they are typically filed at the local police station or sheriff’s office rather than the courthouse.

We abstracted data from all cases received from each state except Florida. In Florida, the large number of case files received (n = 4695) exceeded our available coding resources; therefore, we abstracted data from a random sample of 50% of cases from all counties with greater than 10 ERPO case files based on the case counts by the Office of the State Court Administrator (OSCA). Fifteen Florida counties had a small number (< 10) of cases based on OSCA counts, and we coded all of those. In total, RAs abstracted data from 6,628 ERPO case files (see Table 1) under the 10 domains listed in Table 2 (e.g., criminal legal system; firearm access and possession; and court decisions).

**Table 2:** Domains assessed and examples of variables coded

Petition and petitioner information	State and County Petition Filed In Month and Year Petition Filed Type of Petitioner
Respondent demographics	Gender, Race/Ethnicity, Age Military Status
Suicide Risk^†‡^	Ideation; Threat; Plan; Attempt; Aborted Attempt Method Referenced or Used
Self-harm risk^†‡^	Self-harm, but not a Suicide Attempt Method Used
Risk of violence against others^†‡^	Use of Violence; Threat of Violence Use of Firearm Violence; Threat of Firearm Violence Domestic Violence Restraining Order Mass Shooting Threat
Unlawful or reckless use or brandishing^†‡^	Unlawful or Reckless Use, Display, or Brandishing of a Deadly Weapon Type of Weapon
Risk context^†‡^	Substance/Alcohol Use Harm to Animals State Mental Health Proceedings Mental Illness or Issues Irrational/Erratic Behaviors
Involvement with criminal legal system^†‡^	Arrests Convictions (Level and Type)
Firearm Access and Possession^‡^	Access or Possession at Time Petition Was Filed Recently Acquired Firearms or Ammunition Number & Type of Firearms Possessed or Accessible Number & Type of Firearms Possessed or Accessible Concealed Carry License
Court decisions	Temporary/Ex Parte Order Outcome Final Order Hearing Outcome Reason for Order Denials Firearm Removal or Relinquishment Firearms Returned After Expiration of ERPO

^†^Domain variables indicated or mentioned in petition as ever occurring during lifetime

^‡^Domain variables reported to have occurred as part of precipitating event

### Training and coding procedures

The research team included investigators from nine universities, with members located in each selected state and two additional states. Starting with data collection instruments from two prior ERPO studies (Frattaroli et al. 2020; Zeoli et al. 2021), we collectively developed the data abstraction instrument for the project by comparing the data elements included on each state’s ERPO petition form and the ERPO eligibility criteria listed in each state’s statute against the existing instruments. This process was lengthy due to the vast differences in ERPO petition forms between, and sometimes within, states. The Principal Investigator (PI) and Co-PI curated a list of common and state-specific candidate abstraction variables and shared it with the state PIs and their teams. After the initial draft of the instrument was created, the PI and Co-PI added, removed, edited, and adjusted the items as necessary given feedback from the research team. Through a series of discussions, the multi-state team refined and finalized the list of data elements that comprised the final data collection instrument.

The goal was to create an instrument that would capture the data needed to understand ERPO use. The final instrument had robust sections related to suicide and interpersonal violence risk, among others (see Appendix A in Supplementary material). For suicide risk, we distinguished among ideation, threats, plans, aborted attempts, and attempts where data were available to disambiguate them. For interpersonal violence risk, we abstracted data on threats and uses of violence, separately, with queries capturing the target of the violence or threat. For both suicide and interpersonal violence risk, we captured whether any of the acts or threats of violence involved a firearm. We also included a variable to specify whether these risk behaviors were part of the event that motivated someone to file an ERPO petition (termed the “precipitating event”). Other sections of the data collection instrument specified the risk context of the situation and captured information about substance use, mental health, criminal history, firearm possession or access, and whether a respondent brandished a firearm. Finally, we included sections about ERPO court processes, whether the ERPO was granted, and whether firearms were removed.

State PIs had the option of adding state-specific variables to the instrument and in Maryland, California, and Connecticut, the PIs did. After agreeing on a good working draft of the instrument, we developed training materials that defined each variable and provided examples of coded excerpts from case files and guidance for abstracting the data that the entire research team reviewed, refined, and approved. We then programmed the data collection instrument in Qualtrics, an online survey software program to which all sites had access. Each state PI was then asked to abstract data from a small number of ERPO petitions from their state to ensure suitability of the instrument (the Maryland team was not able to complete this task due to not yet having access to their state’s ERPO casefiles). Feedback was then incorporated into the instrument.

Each state PI staffed their teams according to their state’s volume of ERPO cases. Due to the differences among state’s ERPO petitions and associated forms within the case files, and the need to include RAs on the Institutional Review Board protocol used by their state PIs, we initially planned for each RA to abstract data only from the state they were hired to staff. In practice, some RAs worked across states to manage the variation in access to case files during the study period. Having RAs who were able to code across states allowed us to keep RAs continuously coding even when files were not available in their home states. Specifically, RAs for Maryland and Florida were combined and coded Florida case files while we waited for access to Maryland case files. When Florida was completed, the RAs moved to code Maryland cases. Importantly, RAs coded only one state at a time to avoid introducing errors associated with switching between state case files and differing forms. The project employed 59 RAs over 17 months to code the 6415 cases.

RAs completed didactic training created by the two project PIs via an online learning management system. The training, a mix of videos, readings, and quizzes, included information about ERPOs, the study aims, the data collection instrument and associated definitions, the process for abstracting data, and information about strategies to reduce the risk and impact of secondary trauma. RAs completed the virtual training modules and passed the quizzes before advancing to practice coding two ERPO case files. After coding two case files, RAs participated in two one-hour synchronous sessions hosted by the project PIs to reinforce the online training, give them an opportunity to ask questions, and to review and discuss the test case coding. Once RAs completed these steps, they were cleared by the PIs to code.

The state PIs then trained RAs cleared for coding in the specifics of each state’s case files and variables. The California team held synchronous training sessions until questions had been resolved and RAs felt comfortable proceeding. For Florida and Maryland, RAs attended two virtual synchronous training sessions, one for each state’s ERPO process. In Colorado, RAs were trained using synchronous training sessions and participated in standing biweekly meetings to discuss abstraction issues and element definitions. For Connecticut, the PI developed a state-specific coding manual instructing RAs where to find data elements in the case files. In Washington, RAs were trained using synchronous training sessions and participated in standing weekly meetings to discuss abstraction discrepancies and definitional disagreements.

When coding began in earnest, the process for reaching reliability differed slightly from state to state, depending on the number of RAs and number of cases to be coded. In Washington, for example, a total of 10% of cases were randomly sampled and coded by all RAs to ensure reliability and consistency. In Colorado, 10% of cases were randomly sampled to be double-coded. In Florida, which had the largest number of cases, coding proceeded one county at a time, and RAs double-coded cases until they graduated to single-coder status. For RAs to graduate, they needed to achieve at least a 0.80 inter-rater reliability score. New RAs and those whose scores were below the target were paired with primary RAs (who had reached the 0.80 threshold) until they, too, reached 0.80.

### Data quality and maintaining fidelity to the coding procedures

Because RAs generally coded one state (with the exception of Florida and Maryland RAs), we were unable to quantitatively test reliability of coding between states. Our multiple coding training procedures in which all RAs participated were designed to help ensure consistency. However, due to differences in ERPO documents across states and the lengthy duration of our coding period, it was possible that variations in understanding of variable definitions might have developed among state teams. To combat this possibility, the PI and Co-PI instituted systems to maintain coding pace and consistency among RAs.

Weekly videoconference check-in meetings were implemented, with RAs required to attend at least one meeting each week. Online moderated group chats were used to allow RAs to ask questions as they arose, tagging team members to alert them to the question, enabling them to get answers relatively quickly. The California, Florida, and Maryland teams kept a running document of frequently asked questions that all RAs across states could access during coding. The meetings and group chats served as forums to reinforce training, the coding instrument definitions, troubleshoot coding of complex cases, share consensus with RAs about larger coding questions raised in the online group chats, and develop an inclusive and communicative team dynamic. The check-in meetings and online group chats reduced the number of RA questions needing to be elevated to the PI and Co-PI, maintaining coding pace and consistency.

### Prevention and reduction of secondary trauma

Due to the sometimes detailed and graphic descriptions of crises and violence contained in ERPO case files, there was a risk that RAs would experience secondary trauma through reading them. Secondary trauma, also called vicarious trauma, are the effects of indirect exposure to trauma (McCann and Pearlman 1990). For example, researchers have reported experiencing physical and emotional symptoms (e.g., sleeplessness, an increased awareness of safety) when conducting research on violence and suicide (Mckenzie et al. 2017; Campbell 2002). To minimize the risk of secondary trauma, we instituted protocols to limit RA exposure to cases when needed. For example, the protocol dictated that if an RA decided they could not code a specific case, for any reason, that case was reassigned, no questions asked. By guaranteeing we would not ask for an explanation as to why an RA could not code a case, we allowed them to switch out a case without sharing what might be personal information they did not want to disclose to their supervisors. We also encouraged RAs to shift to completing other study tasks when they needed a break from the intensity of coding. In this way, RAs could request time off from coding case files and shift to completing other research-related tasks until they were ready to re-engage with coding. Additionally, at the weekly check-in meetings, space was held to discuss how RAs were handling the emotional and psychological aspects of coding ERPO case files, cultivating an inclusive and communicative environment where RAs would be comfortable sharing with each other. Importantly, PIs and other meeting leads often began the meetings by sharing what they found emotionally difficult in specific cases to set the tone for the meetings and demonstrate that it is normal to be bothered by the case narratives being read.

Furthermore, the online coding training course completed by all RAs included a module on recognizing signs that might indicate secondary trauma and information on what to do when experiencing such symptoms. A licensed clinical social worker on staff with one of the state teams was available to RAs at some RA meetings and on call for individual appointments, should an RA need it. While the social worker did not establish a therapeutic relationship with RAs, they listened, made suggestions and indicated when it might be necessary to seek other resources to help with the psychological load of coding. Additionally, each state team developed a list of available resources (mainly through their universities, for whom the RAs worked) to which RAs could refer. While this research focused on the possibility that RAs might experience vicarious trauma due to their role in reading and abstracting data from the ERPO casefiles, it is important to recognize that even the most seasoned researcher can experience vicarious trauma and benefit from the steps detailed here.

## Discussion

By coordinating data collection on ERPO cases across states, we efficiently achieved greater explanatory power through pooled analyses and direct comparisons than would be possible if we had examined ERPO use in each of these states independently. Analyzing the breadth of violence risks and contexts in which the risks occur in ERPO case files requires attention to detail and standard data collection protocols to be in place and followed. Considering ERPO petitions describe the ways in which the respondent is at risk of harming themselves and/or others, and therefore can contain graphic descriptions of violence and threats (including mass shooting threats, suicide attempts, and domestic violence) conducting research about ERPOs carries risks of secondary trauma. This account of our processes can inform future firearm violence prevention research by providing a reference for how to undertake similar projects in terms of data acquisition, coding, data quality, and strategies to promote health wellness among RAs.

The study used cross-sectional administrative data. Relying on administrative data meant that the processes described are for coding data reported in the case files only. We did not seek out information beyond what was provided (typically solely from the petitioner's perspective) through the ERPO case files. We note that the structure and level of information available in the case files varied across and within states, as well as between petitioner types (law enforcement or civilian). Comparisons of ERPO use across states requires consideration of this variability. In states where law enforcement officers are the only authorized petitioners, information reported about respondents and precipitating events followed a relatively uniform reporting style, although the narrative style of these reports meant that the content was not uniformly consistent in relation to the data points to be abstracted. Where civilians, mainly family members and intimate partners, were authorized to petition, the presentation and type of information included in the petitions varied more significantly.

To our knowledge, this study is the first of its kind to analyze a multi-state sample of ERPOs. The process of standardizing information and abstracting data across states consistently to describe state-level ERPO implementation and assess impacts of the law offers researchers some insight into what such an undertaking involves and provides a foundation on which to interpret findings reported from the six-state study.

## Supplementary Information

Supplementary Material 1.

## Data Availability

A limited dataset generated from ERPO case files will be available at ICPSR upon publication of research from the multi-state study.

## Abbreviations

ERPO: Extreme risk protection order
RA: Research assistant
DOJ: Department of Justice
CCIS: Comprehensive case information system
PI: Principal investigator
