# Supplementary material for: A multi-state evaluation of extreme risk protection orders: a research protocol
Source: Inj Epidemiol. 2024 Sep 9;11:49. doi: 10.1186/s40621-024-00535-z (PMC11382528; doi:10.1186/s40621-024-00535-z)
Supplement: Supplementary file 1 — Supplementary Material 1. [file 40621_2024_535_MOESM1_ESM.docx]

Appendix A

ERPO Data Collection Instrument

ERPO data collection instrument – Feb 23, 2021

**Start of Block: Coding info**

Coder 1 Coder's initials

- Coder's initials (1)


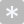


Coder 2 Date this petition was coded


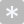


Coder 3 Case ID (2 letter state abbreviation + 4 numbers representing order petition was given a case ID in. For example, the 8th Florida petition given a case ID is FL0008.)


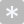


Coder 4 Respondent ID (A 5-digit unique number in which the first digit indicates the state and the remaining four are sequence numbers from 0001 through 9999)


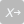


Coder 5 Which documents were included in the case file? (click all that apply)

▢ ERPO petition (1)

▢ Affidavit or other supporting docs (2)

▢ Emergency order/Interim order (3)

▢ Temporary (ex parte) order / Risk warrant if CT (4)

▢ Final order (5)

▢ Respondent served temporary ERPO (6)

▢ Respondent served full ERPO (7)

▢ Respondent's motion to vacate order (9)

▢ Hearing to vacate order (10)

▢ Petitioner's motion to renew order (11)

▢ Hearing to renew order (12)

▢ Continuance (13)

▢ Document regarding firearms (14)

▢ Court transaction sheet (15)

▢ Other orders noted in the file (list and state whether any include a firearm prohibition) (16)

▢ Other documents included in the file (17)

**End of Block: Coding info**

**Start of Block: Petition/Petitioner Information**

Info 1 State

▼ California (1) ... Washington (6)


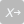


*Display This Question:*

*If State = California*

Info 2 California County

▼ Alameda (1) ... Yuba (58)

*Display This Question:*

*If State = Colorado*

Info 3 Colorado County

▼ Adams (1) ... Yuma (64)

*Display This Question:*

*If State = Connecticut*

Info 4 Connecticut County

▼ Fairfield (1) ... Windham (8)

*Display This Question:*

*If State = Florida*

Info 5 Florida County

▼ Alachua (1) ... Washington (67)

*Display This Question:*

*If State = Maryland*

Info 6 Maryland County

▼ Allegany (1) ... Worcester (24)

*Display This Question:*

*If State = Washington*

Info 7 Washington County

▼ Adams (1) ... Yakima (39)

Info 8 Month the petition was filed with the court.

▼ January (1) ... December (12)

*Display This Question:*

*If State = California*

Info 9 Year the petition was filed with the court.

▼ 2020 (1) ... 2016 (5)

*Display This Question:*

*If State = Colorado*

Info 10 Year the petition was filed with the court.

▼ 2020 (1) ... 2020 (1)

*Display This Question:*

*If State = Connecticut*

Info 11 Year the petition was filed with the court.

▼ 2020 (1) ... 2012 (9)

*Display This Question:*

*If State = Florida*

Info 12 Year the petition was filed with the court.

▼ 2020 (1) ... 2018 (3)

*Display This Question:*

*If State = Maryland*

Info 13 Year the petition was filed with the court.

▼ 2020 (1) ... 2018 (3)

*Display This Question:*

*If State = Washington*

Info 14 Year the petition was filed with the court.

▼ 2020 (1) ... 2016 (5)


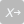


Info 15 Type of petitioner.

- Law enforcement (1)
- Immediate family (non-intimate partner) (2)
- Intimate partner (spouse, dating partner, shared child) (3)
- Health professional (4)
- Other: (5)
- Unknown (6)


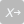


*Display This Question:*

*If Type of petitioner. = Law enforcement*

Info 16 If a law enforcement petitioner, who alerted law enforcement to concerns about the respondent? (e.g., who called the police?) (click all that apply)

▢ Immediate family (non-intimate partner) (1)

▢ Intimate partner (spouse, dating partner, shared child) (2)

▢ Friend (3)

▢ Self (4)

▢ Health professional (5)

▢ Employer/Employee/Coworker (6)

▢ Other: (7)

▢ Unknown (8)

Info 17 What is the name of the judge on the case? (write "illegible" if you cannot read the signature)

**End of Block: Petition/Petitioner Information**

**Start of Block: Respondent Demographics**

Demo 1 Respondent gender

- Male (1)
- Female (2)
- Other (3)
- Unknown (4)

Demo 2 Race/ethnicity of respondent. (Click all that apply)

▢ White (1)

▢ Black or African American (2)

▢ American Indian or Alaska Native (3)

▢ Asian (4)

▢ Native Hawaiian or Pacific Islander (5)

▢ Latino/Latina/Latinx or Hispanic (6)

▢ Other (7)

▢ Unknown (8)

Demo 3 Age of respondent

- Date of birth (MM/DD/YYYY) (1)
- Age in years - use this link to calculate age if needed (2)


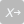


Demo 4 Is respondent a minor (age 17 or younger)?

▼ Yes (1) ... Unknown (2)


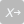


Demo 5 Does the file indicate that the respondent is currently or was formerly in the armed services (military)?

▼ Yes (1) ... No (0)

**End of Block: Respondent Demographics**

**Start of Block: Suicide risk**


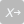


Suicide 1 Is there information in this file that suggests the respondent is suicidal?

▼ Yes (1) ... No (0)

*Display This Question:*

*If Is there information in this file that suggests the respondent is suicidal? = Yes*

Suicide 2 What suicide risk behavior(s) was the respondent reported to have engaged in? (check all that apply - if checking "no specific information" that should be the only option marked)

▢ Suicide Ideation (1)

▢ Suicide Threats (2)

▢ Suicide Plans (3)

▢ Aborted Suicide Attempts (4)

▢ Suicide Attempts (5)

▢ ⊗No specific information on suicidality is included in the file (6)


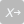


*Display This Question:*

*If What suicide risk behavior(s) was the respondent reported to have engaged in? (check all that app... = Suicide Ideation*

Suicide 3 What method of suicide are the respondent's IDEATIONS of suicide reported to include? (click all that apply)

▢ Firearm (1)

▢ Sharp instrument (2)

▢ Asphyxiation (3)

▢ Poisoning or overdose (4)

▢ Jump/fall from great height (5)

▢ Suicide by cop (6)

▢ Other (7)

▢ Unknown (8)


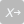


*Display This Question:*

*If What suicide risk behavior(s) was the respondent reported to have engaged in? (check all that app... = Suicide Threats*

Suicide 4 What method of suicide are the respondent's THREATS of suicide reported to include? (click all that apply)

▢ Firearm (1)

▢ Sharp instrument (2)

▢ Asphyxiation (3)

▢ Poisoning or overdose (4)

▢ Jump/fall from great height (5)

▢ Suicide by cop (6)

▢ Other (7)

▢ Unknown (8)


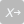


*Display This Question:*

*If What suicide risk behavior(s) was the respondent reported to have engaged in? (check all that app... = Suicide Plans*

Suicide 5 What method of suicide are the respondent's PLANS to die by suicide reported to include? (click all that apply)

▢ Firearm (1)

▢ Sharp instrument (2)

▢ Asphyxiation (3)

▢ Poisoning or overdose (4)

▢ Jump/fall from great height (5)

▢ Suicide by cop (6)

▢ Other (7)

▢ Unknown (8)


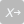


*Display This Question:*

*If What suicide risk behavior(s) was the respondent reported to have engaged in? (check all that app... = Aborted Suicide Attempts*

Suicide 6 What method of suicide are the respondent's ABORTED suicide attempts reported to include? (click all that apply)

▢ Firearm (1)

▢ Sharp instrument (2)

▢ Asphyxiation (3)

▢ Poisoning or overdose (4)

▢ Jump/fall from great height (5)

▢ Suicide by cop (6)

▢ Other (7)

▢ Unknown (8)


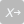


*Display This Question:*

*If What suicide risk behavior(s) was the respondent reported to have engaged in? (check all that app... = Suicide Attempts*

Suicide 7 What method of suicide are the respondent's suicide ATTEMPTS reported to include? (click all that apply)

▢ Firearm (1)

▢ Sharp instrument (2)

▢ Asphyxiation (3)

▢ Poisoning or overdose (4)

▢ Jump/fall from great height (5)

▢ Suicide by cop (6)

▢ Other (7)

▢ Unknown (8)


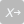


*Display This Question:*

*If Is there information in this file that suggests the respondent is suicidal? = Yes*

Suicide 8 Were suicide ideations, threats, plans, aborted attempts, or attempts reported to be part of the precipitating event? (click all that apply)

▢ Suicide IDEATIONS were part of the precipitating event (1)

▢ Suicide THREATS were part of the precipitating event (2)

▢ Suicide PLANS were part of the precipitating event (3)

▢ ABORTED suicide attempts were part of the precipitating event (4)

▢ Suicide ATTEMPTS were part of the precipitating event (5)

▢ No ideations, threats, or attempts were part of the precipitating event (6)


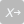


*Display This Question:*

*If Were suicide ideations, threats, plans, aborted attempts, or attempts reported to be part of the... != No ideations, threats, or attempts were part of the precipitating event*

*And What method of suicide are the respondent's IDEATIONS of suicide reported to include? (click all... = Firearm*

*Or What method of suicide are the respondent's THREATS of suicide reported to include? (click all th... = Firearm*

*Or What method of suicide are the respondent's PLANS to die by suicide reported to include? (click a... = Firearm*

*Or What method of suicide are the respondent's ABORTED suicide attempts reported to include? (click... = Firearm*

*Or What method of suicide are the respondent's suicide ATTEMPTS reported to include? (click all that... = Firearm*

Suicide 9 Was a firearm reported to be the method of suicide in the ideations, threats, plans, aborted attempts, or attempts that were part of the precipitating event?

▼ Yes (1) ... No (0)

**End of Block: Suicide risk**

**Start of Block: Self-harm risk**


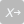


Harm 1 Is there information in the ERPO filing that indicates that the respondent self-harmed in a way that was not a suicide attempt?

- Yes (1)
- No (0)


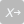


*Display This Question:*

*If Is there information in the ERPO filing that indicates that the respondent self-harmed in a way t... =*

*Yes*

Harm 2 What weapon was used?

▢ Firearm (1)

▢ Bodily weapon (2)

▢ Other weapon (3)

▢ Unknown (4)


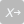


*Display This Question:*

*If Is there information in the ERPO filing that indicates that the respondent self-harmed in a way t... =*

*Yes*

Harm 3 What self-harm (not suicide attempt) weapon was used as part of the precipitating event? (click all that apply)

▢ Firearm (1)

▢ Bodily weapon (2)

▢ Other weapon (3)

▢ Unknown (4)

▢ No self-harm was part of the precipitating event (5)

**End of Block: Self-harm risk**

**Start of Block: Violence against others**


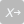


Viol 1 Is there information in the file to indicate respondent's risk of violence against others, either through use or threat of physical force, violence, or harm against another person? (click all that apply)

▢ There is information on the USE of violence (1)

▢ There is information on THREATS of violence (2)

▢ There is no information on threats or uses of violence (3)

Viol 2 Is there information in the file to indicate the respondent was or is currently under a domestic violence restraining order (DVRO) (also called a protection order, stay away order, injunction, or no contact order)?

▼ Respondent is or was under a DVRO at some point (1) ... There is no evidence that respondent was ever under a DVRO (3)

*Display This Question:*

*If Is there information in the file to indicate respondent's risk of violence against others, either... = There is information on the USE of violence*


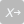


Viol 3 Who was the apparent target of the USE of violence, according to the case file? (click all that apply)

▢ Intimate partner (1)

▢ Adult family members (excluding intimate partners) (2)

▢ Family members under age 18 (excluding intimate partners) (3)

▢ Family members of unknown age (excluding intimate partners) (4)

▢ Family member or intimate partner (unclear which) (5)

▢ Minor (6)

▢ Law enforcement (7)

▢ Other targets (be careful to detail description of target(s) as clearly as possible)

(8)

▢ Person or group is unclear (9)

▢ Hallucination (10)


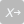


*Display This Question:*

*If Is there information in the file to indicate respondent's risk of violence against others, either... = There is information on the USE of violence*

Viol 4 If the USE of violence was reported to have occurred in the PRECIPITATING event, who was the apparent target of that use of violence? (click all that apply)

▢ Intimate partner (1)

▢ Adult family members (excluding intimate partners) (2)

▢ Family members under age 18 (excluding intimate partners) (3)

▢ Family members of unknown age (excluding intimate partners) (4)

▢ Family member or intimate partner (unclear which) (5)

▢ Minor (6)

▢ Law enforcement (7)

▢ Other targets (be careful to detail description of target(s) as clearly as possible)

(8)

▢ Person or group is unclear (9)

▢ Hallucination (10)

▢ ⊗Use of violence was not part of the precipitating event (11)


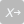


*Display This Question:*

*If Is there information in the file to indicate respondent's risk of violence against others, either... = There is information on the USE of violence*

Viol 5 According to the case file, which of these were apparent targets of the USE of *firearm*

violence, if any? (click which people/groups were the targets of firearm violence)

▢ Intimate partner (1)

▢ Adult family members (excluding intimate partners) (2)

▢ Family members under age 18 (excluding intimate partners) (3)

▢ Family members of unknown age (excluding intimate partners) (4)

▢ Family member or intimate partner (unclear which) (5)

▢ Minor (6)

▢ Law enforcement (7)

▢ Other targets (be careful to detail description of target(s) as clearly as possible)

(8)

▢ Person or group is unclear (9)

▢ Hallucination (10)

▢ ⊗No firearm uses were reported (11)


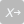


*Display This Question:*

*If According to the case file, which of these were apparent targets of the USE of firearm violence,... != No firearm uses were reported*

*And Is there information in the file to indicate respondent's risk of violence against others, either... = There is information on the USE of violence*

Viol 6 If the USE of *firearm* violence was reported to have occurred in the PRECIPITATING event, who was the apparent target of that use of firearm violence? (click all that apply)

▢ Intimate partner (1)

▢ Adult family members (excluding intimate partners) (2)

▢ Family members under age 18 (excluding intimate partners) (3)

▢ Family members of unknown age (excluding intimate partners) (4)

▢ Family member or intimate partner (unclear which) (5)

▢ Minor (6)

▢ Law enforcement (7)

▢ Other targets (be careful to detail description of target(s) as clearly as possible)

(8)

▢ Person or group is unclear (9)

▢ Hallucination (10)

▢ ⊗No firearm uses were reported in the precipitating event (11)


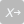


*Display This Question:*

*If Is there information in the file to indicate respondent's risk of violence against others, either... = There is information on THREATS of violence*

Viol 7 Who was the apparent target of THREATS of violence, according to the case file? (click all that apply)

▢ Intimate partner (1)

▢ Adult family members (excluding intimate partners) (2)

▢ Family members under age 18 (excluding intimate partners) (3)

▢ Family members of unknown age (excluding intimate partners) (4)

▢ Family member or intimate partner (unclear which) (5)

▢ Minor (6)

▢ Law enforcement (7)

▢ Other targets (be careful to detail description of target(s) as clearly as possible)

(8)

▢ Person or group is unclear (9)

▢ Hallucination (10)


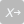


*Display This Question:*

*If Is there information in the file to indicate respondent's risk of violence against others, either... = There is information on THREATS of violence*

Viol 8 If the THREAT of violence was reported to have occurred in the PRECIPITATING event, who was the apparent target of that threat of violence? (click all that apply)

▢ Intimate partner (1)

▢ Adult family members (excluding intimate partners) (2)

▢ Family members under age 18 (excluding intimate partners) (3)

▢ Family members of unknown age (excluding intimate partners) (4)

▢ Family member or intimate partner (unclear which) (5)

▢ Minor (6)

▢ Law enforcement (7)

▢ Other targets (be careful to detail description of target(s) as clearly as possible)

(8)

▢ Person or group is unclear (9)

▢ Hallucination (10)

▢ ⊗Threats of violence were not part of the precipitating event (11)


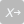


*Display This Question:*

*If Is there information in the file to indicate respondent's risk of violence against others, either... = There is information on THREATS of violence*

Viol 9 According to the case file, which of these apparent targets of THREATS were threatened with *firearm* violence? (click which people/groups were the targets of threatened firearm violence)

▢ Intimate partner (1)

▢ Adult family members (excluding intimate partners) (2)

▢ Family members under age 18 (excluding intimate partners) (3)

▢ Family members of unknown age (excluding intimate partners) (4)

▢ Family member or intimate partner (unclear which) (5)

▢ Minor (6)

▢ Law enforcement (7)

▢ Other targets (be careful to detail description of target(s) as clearly as possible)

(8)

▢ Person or group is unclear (9)

▢ Hallucination (10)

▢ ⊗No firearm threats were reported (11)

*Display This Question:*

*If According to the case file, which of these apparent targets of THREATS were threatened with firea... != No firearm threats were reported*

*And Is there information in the file to indicate respondent's risk of violence against others, either... = There is information on THREATS of violence*

Viol 10 If the THREAT of *firearm* violence was reported to have occurred in the PRECIPITATING event, who was the apparent target of that use of firearm violence? (click all that apply)

▢ Intimate partner (1)

▢ Adult family members (excluding intimate partners) (2)

▢ Family members under age 18 (excluding intimate partners) (3)

▢ Family members of unknown age (excluding intimate partners) (4)

▢ Family member or intimate partner (unclear which) (5)

▢ Minor (6)

▢ Law enforcement (7)

▢ Other targets (be careful to detail description of target(s) as clearly as possible)

(8)

▢ Person or group is unclear (9)

▢ Hallucination (10)

▢ ⊗No firearm threats were reported as part of the precipitating event (11)

*Display This Question:*

*If According to the case file, which of these were apparent targets of the USE of firearm violence,... != No firearm uses were reported*

*Or According to the case file, which of these apparent targets of THREATS were threatened with firea... != No firearm threats were reported*

Viol 11 Is the respondent reported to have threatened a mass shooting?

▼ Yes (1) ... No (0)


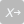


*Display This Question:*

*If Is the respondent reported to have threatened a mass shooting? = Yes*

Viol 12 Was the report mass shooting threatened to take place in a public or private venue? (click all that apply)

▢ Public (1)

▢ Private (2)

▢ Unknown (3)


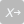


*Display This Question:*

*If Is the respondent reported to have threatened a mass shooting? = Yes*

Viol 13 Was the reported mass shooting threat part of the PRECIPITATING event?

▼ Yes (1) ... Not reported (2)

*Display This Question:*

*If Is the respondent reported to have threatened a mass shooting? = Yes*

Viol 14 Please describe the mass shooting threat.

**End of Block: Violence against others**

**Start of Block: Risk to self or others**


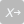


*Display This Question:*

*If Is there information in this file that suggests the respondent is suicidal? = No*

*And Is there information in the ERPO filing that indicates that the respondent self-harmed in a way t...*

*= No*

*And Is there information in the file to indicate respondent's risk of violence against others, either... = There is no information on threats or uses of violence*

Q120 Does the petition form indicate that there was a risk of harm to self or others, but there is no additional information in the case specifying if there was a risk of harm to self, a risk of harm to others, or both?

▼ Yes (1) ... No (0)

**End of Block: Risk to self or others**

**Start of Block: Firearm brandish risk**


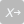


Firearm 1 Is there information in the file to indicate the unlawful or reckless use, display, or brandishing of a deadly weapon?

▼ Yes (1) ... No (0)


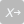


*Display This Question:*

*If Is there information in the file to indicate the unlawful or reckless use, display, or brandishin... =*

*Yes*

Firearm 2 What deadly weapon was used? (click all that apply)

▢ Firearm (1)

▢ Other Weapon (2)

▢ Unknown (3)


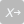


*Display This Question:*

*If Is there information in the file to indicate the unlawful or reckless use, display, or brandishin... =*

*Yes*

Firearm 3 What deadly weapon, if any, was unlawfully or recklessly used, displayed, or brandished as part of the precipitating event, according to the case file? (click all that apply)

▢ Firearm (1)

▢ Other weapon (2)

▢ Unknown weapon (3)

▢ No deadly weapon was unlawfully or recklessly used in the precipitating event. (4)

**End of Block: Firearm brandish risk**

**Start of Block: Risk context**


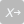


Risk 1 Is substance/alcohol use by the respondent mentioned in the petition? (click all that apply, but these are mutually exclusive)

▢ Illicit drugs (except heroin). Does not include use or misuse of prescription drugs or marijuana (1)

▢ Opioid use (including heroin). Includes any prescription opioids and/or heroin (2)

▢ Marijuana (3)

▢ Vague reference to drugs (4)

▢ Other substance misuse (5)

▢ Alcohol (6)

▢ Drugs or alcohol misused, but unclear which one (7)

▢ No substance/alcohol use mentioned (8)


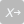


*Display This Question:*

*If Is substance/alcohol use by the respondent mentioned in the petition? (click all that apply, but... != No substance/alcohol use mentioned*

Risk 2 Which substances/alcohol use does the file mention were used by the respondent during the PRECIPITATING event, if any? (click all that apply)

▢ Illicit drugs (except heroin). Does not include use or misuse of prescription drugs or marijuana (1)

▢ Opioid use (including heroin). Includes any prescription opioids and/or heroin (2)

▢ Marijuana (3)

▢ Vague reference to drugs (4)

▢ Other substance misuse (5)

▢ Alcohol (6)

▢ No substance/alcohol use mentioned during the PRECIPITATING event (7)


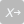


Risk 3 Is there mention of the respondent harming animals?

▼ Yes (1) ... No (0)


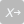


*Display This Question:*

*If Is there mention of the respondent harming animals? = Yes*

Risk 4 Did the respondent harm animals as part of the PRECIPITATING event?

▼ Yes (1) ... Unknown (2)


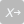


Risk 5 Was the respondent subject to state proceedings regarding mental health concerns?

▼ Yes (1) ... No (0)


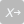


*Display This Question:*

*If Was the respondent subject to state proceedings regarding mental health concerns? = Yes*

Risk 6 Were the state proceedings regarding mental health concerns part of the PRECIPITATING event, as reported in the case file.

▼ Yes (1) ... Unclear (2)


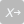


Risk 7 Does the case file include an indication that the respondent has a mental illness, recurring mental health issue, is neurodiverse, in cognitive decline, or engaged in irrational/erratic behaviors?

▼ Yes (1) ... No (0)

*Display This Question:*

*If Does the case file include an indication that the respondent has a mental illness, recurring ment... =*

*Yes*

Risk 8 If yes, click all that apply:

▢ There is a specific mental health diagnosis listed, a statement that there is a mental health diagnosis, or a psychiatric medication listed (assume diagnosis required to be prescribed medication), or there is evidence the person experienced a psychiatric

hospitalization. List diagnosis (1)

▢ There is a vague statement about mental illness or other vague mental health phrases (2)

▢ The respondent is reported to have engaged in behaviors that are unpredictable, do not seem to make sense, are out of character, or outside the norms of behavior (irrational or erratic behaviors) (3)

▢ The respondent is neurodiverse (4)

▢ The respondent has cognitive decline from a traumatic brain injury (5)

▢ The respondent has age-related cognitive decline (6)

▢ The respondent is impaired from a medical illness other than dementia or Alzheimer's (7)

▢ The respondent has impaired cognitive abilities from an unstated cause (8)


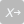


*Display This Question:*

*If Does the case file include an indication that the respondent has a mental illness, recurring ment... =*

*Yes*

Risk 9 Were irrational/erratic behaviors part of the PRECIPITATING event, according to the case file?

▼ Yes (1) ... Unclear (2)

**End of Block: Risk context**

**Start of Block: Criminal history**


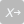


Crime 1 Is there information in the file indicating the respondent has a criminal history? (Note: This question refers to the respondent's lifetime.)

▼ Yes (1) ... No (0)


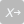


*Display This Question:*

*If Is there information in the file indicating the respondent has a criminal history? (Note: This qu... =*

*Yes*

Crime 2 If there is direct information about a **specific arrest** during the respondent's lifetime, indicate what the respondent has been arrested for. (click all the apply)

▢ Violence against an intimate partner or family member (1)

▢ Violence against someone other than an intimate partner or family member (2)

▢ Stalking (3)

▢ Crime related to illicit substances (4)

▢ Driving under the influence (5)

▢ A crime involving a firearm (6)

▢ Cruelty to animals (7)

▢ Other crime (nonviolent) (8)

▢ Arrested for unknown crime (9)

▢ ⊗No information on a specific arrest is in the file (10)

*Display This Question:*

*If Is there information in the file indicating the respondent has a criminal history? (Note: This qu... =*

*Yes*

*And If there is direct information about a specific arrest during the respondent's lifetime, indicate... != No information on a specific arrest is in the file*


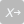


Crime 3 Is there evidence in the case file that the respondent was arrested during the PRECIPITATING event?

▼ Yes (1) ... No (0)


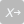


*Display This Question:*

*If Is there information in the file indicating the respondent has a criminal history? (Note: This qu... =*

*Yes*

Crime 4 Is there information in the file indicating the respondent had been convicted of a crime in their lifetime?

▼ Yes (1) ... No (0)


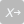


*Display This Question:*

*If Is there information in the file indicating the respondent had been convicted of a crime in their... =*

*Yes*

Crime 5 According to the case file, was the respondent convicted for something that occurred during the PRECIPITATING event?

▼ Yes (1) ... Unknown (2)


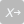


*Display This Question:*

*If Is there information in the file indicating the respondent had been convicted of a crime in their... =*

*Yes*

Crime 6 According to the case file, at what level has the respondent been convicted in their lifetime? (click all that apply)

▢ Felony (1)

▢ Misdemeanor (2)

▢ Ordinance violation (3)

▢ Unknown (4)


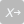


*Display This Question:*

*If Is there information in the file indicating the respondent had been convicted of a crime in their... =*

*Yes*

Crime 7 If known, indicate what the respondent has been convicted for in their lifetime. (click all the apply)

▢ Violence against an intimate partner or family member (1)

▢ Violence against someone other than an intimate partner or family member (2)

▢ Stalking (3)

▢ Crime related to illicit substances (4)

▢ Driving under the influence (5)

▢ A crime involving a firearm (6)

▢ Cruelty to animals (7)

▢ Other crime (nonviolent) (8)

▢ Convicted for an unknown crime (9)

*Display This Question:*

*If If known, indicate what the respondent has been convicted for in their lifetime. (click all the a... = Violence against an intimate partner or family member*

Crime 8 If known, indicate whether the respondent was convicted for violence against an intimate partner or for violence against a non-intimate family member. (click all that apply if convicted for violence against intimate and violence against a family member)

▢ Intimate partner (1)

▢ Non-intimate family member (2)

▢ Unclear if intimate partner or family member (3)

**End of Block: Criminal history**

**Start of Block: Gun possession**


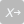


Poss 1 According to the case file, did the respondent possess a firearm at the time the petition was filed? (click all that apply)

▢ There is evidence in the file of firearm possession (1)

▢ Respondent was recently dispossessed as part of the ERPO petition process (2)

▢ Respondent was dispossessed by law enforcement before the precipitating event (3)

▢ Respondent recently gave their firearms to someone else (4)

▢ ⊗Respondent does not posses or have access to a firearm (5)

▢ Respondent has access to, but does not possess, a firearm (6)

▢ ⊗It is unknown whether the respondent possessed a firearm (7)


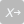


*Display This Question:*

*If According to the case file, did the respondent possess a firearm at the time the petition was fil... = There is evidence in the file of firearm possession*

*Or According to the case file, did the respondent possess a firearm at the time the petition was fil... = Respondent was recently dispossessed as part of the ERPO petition process*

*Or According to the case file, did the respondent possess a firearm at the time the petition was fil... = Respondent was dispossessed by law enforcement before the precipitating event*

*Or According to the case file, did the respondent possess a firearm at the time the petition was fil... = Respondent recently gave their firearms to someone else*

*Or According to the case file, did the respondent possess a firearm at the time the petition was fil... = Respondent has access to, but does not possess, a firearm*

Poss 2 Does the petition indicate how many firearms the respondent possessed or was able to access before the petition was filed (including the firearms that were dispossessed or given to someone else)?

▼ Yes (1) ... No (0)

*Display This Question:*

*If Does the petition indicate how many firearms the respondent possessed or was able to access befor... = Yes*

Poss 3 How many firearms did the respondent possess or have access to (including the firearms that were dispossessed or given to someone else)?

- Fill in a number (1)

*Display This Question:*

*If According to the case file, did the respondent possess a firearm at the time the petition was fil... = There is evidence in the file of firearm possession*

*Or According to the case file, did the respondent possess a firearm at the time the petition was fil... = Respondent was recently dispossessed as part of the ERPO petition process*

*Or According to the case file, did the respondent possess a firearm at the time the petition was fil... = Respondent was dispossessed by law enforcement before the precipitating event*

*Or According to the case file, did the respondent possess a firearm at the time the petition was fil... = Respondent recently gave their firearms to someone else*

*Or According to the case file, did the respondent possess a firearm at the time the petition was fil... = Respondent has access to, but does not possess, a firearm*

Poss 4 What type of firearms? (Click all that apply)

▢ Handgun (1)

▢ Long gun (2)

▢ Unknown (3)


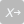


Poss 5 Is there information in the file indicating that the respondent has recently acquired firearms or ammunition?

▼ Respondent possessed a recently acquired firearm or ammunition (1) ... No information in file about purchase or acquisitions of firearms or ammunition (4)


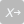


Poss 6 Is there information indicating that the respondent has a concealed carry license?

▼ Yes (1) ... No (0)

**End of Block: Gun possession**

**Start of Block: Court decisions**


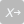


Court 1 Is there information suggesting the respondent is already under an existing extreme risk protection order?

▼ Yes (1) ... No (0)


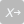


Court 2 Is there information indicating the respondent violated a current or previous ERPO?

▼ Yes (1) ... No (0)


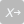


Court 3 Did the petitioner request an ex parte (temporary) ERPO?

▼ Yes (1) ... Unknown (2)

*Display This Question:*

*If Did the petitioner request an ex parte (temporary) ERPO? = Yes*

Court 4 If requested, what was the outcome of the ex parte (temporary) hearing?

▼ Granted (1) ... Unknown (4)

*Display This Question:*

*If If requested, what was the outcome of the ex parte (temporary) hearing? = Denied*

Court 5 If denied, what was the reason for denial? (click all that apply)

▢ Petitioner not covered in statute (1)

▢ Petitioner did not attend hearing (2)

▢ Evidence did not meet statutory standard (3)

▢ Other (4)

▢ Unknown (5)


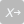


*Display This Question:*

*If If requested, what was the outcome of the ex parte (temporary) hearing? = Granted*

Court 6 Is there indication in the file that there was a continuance?

▼ Yes (1) ... No (0)

*Display This Question:*

*If Is there indication in the file that there was a continuance? = Yes*

Court 7 How many continuances are in the file?

- Indicate number of continuances (1)

*Display This Question:*

*If Is there indication in the file that there was a continuance? = Yes*

Court 8 How many days was the ex parte order continued for?

- Indicate number of days - use this link to calculate number of days (1)


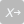


Court 9 Was there a final order hearing? (this includes cases in which there was a stipulation to the final order)

▼ Yes (1) ... Unknown (3)


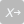


*Display This Question:*

*If Was there a final order hearing? (this includes cases in which there was a stipulation to the fin... =*

*Yes*

Court 10 Was the respondent represented by an attorney at the final hearing?

▼ Respondent was represented by an attorney (1) ... Unknown (2)


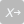


*Display This Question:*

*If Was there a final order hearing? (this includes cases in which there was a stipulation to the fin... =*

*Yes*

Court 11 Was the petitioner represented by an attorney at the final hearing?

▼ Petitioner was represented by an attorney (1) ... Unknown (2)

*Display This Question:*

*If Was there a final order hearing? (this includes cases in which there was a stipulation to the fin... =*

*Yes*

Court 12 If known, how many days were there between the ex parte decision and the final order hearing?

- Indicate number of days - use this link to calculate number of days (1)

*Display This Question:*

*If Was there a final order hearing? (this includes cases in which there was a stipulation to the fin... =*

*Yes*

Court 13 What was the outcome of the final order hearing?

▼ Granted (1) ... Other (5)

*Display This Question:*

*If What was the outcome of the final order hearing? = Denied*

Court 14 If denied, what was the reason for the denial? (click all that apply)

▢ Petitioner not covered in the statute (1)

▢ Petitioner did not attend hearing (2)

▢ Evidence did not meet statutory standard (3)

▢ Other (4)

▢ Unknown (5)


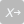


*Display This Question:*

*If What was the outcome of the final order hearing? = Granted*

*Or What was the outcome of the final order hearing? = Granted - the Respondent stipulated to the final order*

Court 15 As part of the final order granting, was the respondent ordered to have a mental health evaluation?

▼ Yes (1) ... Unknown (2)


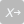


*Display This Question:*

*If What was the outcome of the final order hearing? = Granted*

*And What was the outcome of the final order hearing? = Granted - the Respondent stipulated to the final order*

Court 16 As part of the final order granting, was the respondent ordered to have a chemical dependency evaluation?

▼ Yes (1) ... Unknown (2)

*Display This Question:*

*If What was the outcome of the final order hearing? = Granted*

*Or What was the outcome of the final order hearing? = Granted - the Respondent stipulated to the final order*

*Or What was the outcome of the final order hearing? = Unknown Or What was the outcome of the final order hearing? = Other*


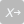


Court 17 Did the file include a motion to vacate the ERPO?

▼ Yes (1) ... No (0)


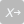


*Display This Question:*

*If Did the file include a motion to vacate the ERPO? = Yes*

Court 18 Was the motion to vacate granted?

▼ Yes (1) ... Unknown (2)


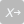


*Display This Question:*

*If What was the outcome of the final order hearing? = Granted*

*Or What was the outcome of the final order hearing? = Granted - the Respondent stipulated to the final order*

*Or What was the outcome of the final order hearing? = Unknown Or What was the outcome of the final order hearing? = Other*

Court 19 Did the file include a motion to renew the ERPO?

▼ Yes (1) ... No (0)


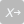


*Display This Question:*

*If Did the file include a motion to renew the ERPO? = Yes*

Court 20 Was the motion to renew granted?

▼ Yes (1) ... Unknown (2)


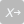


Court 21 Is there information in the file indicating that firearms were removed or relinquished after an ERPO was granted?

▼ Yes (1) ... No (0)


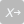


*Display This Question:*

*If Is there information in the file indicating that firearms were removed or relinquished after an E... =*

*Yes*

Court 22 Is there information in the file indicating the respondent threatened or harmed anyone during the process of removing or relinquishing firearms after an ERPO was granted?

- Yes, please describe (1)
- No (0)


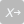


*Display This Question:*

*If Is there information in the file indicating that firearms were removed or relinquished after an E... =*

*Yes*

Court 23 Is there information in the file indicating that firearms were returned after the ERPO was no longer in place?

▼ Yes (1) ... No (0)

*Display This Question:*

*If Is there information in the file indicating that firearms were returned after the ERPO was no lon... =*

*Yes*

Court 24 If there is information in the file indicating how long firearms were held by law enforcement, indicate how long. In other words, how long between removal and return of firearms? Choose either days, weeks, or months as the time unit and approximate as best you can. Use this link to calculate time if needed.

- Days (1)
- Weeks (2)
- Months (3)
- Unable to estimate from information in the file (4)

**End of Block: Court decisions**

**Start of Block: COVID and final Qs**

Final 1 Is there any information in the report suggesting that COVID-19's impacts played a role in this petition? If yes, please describe

Final 2 Were you unsure of how to code any of the questions? If so, please describe the problem here and how you made your coding decision.

Final 3 If this is a particularly interesting or exemplary case, explain why.

**End of Block: COVID and final Qs**
